# Enhanced Synovial Fluid Rheology in Moderate Knee Osteoarthritis Through Combined Intra-Articular Hyaluronic Acid and Multimodal Physiotherapy: A Monocentric Observational Study

**DOI:** 10.3390/jcm14176051

**Published:** 2025-08-27

**Authors:** Daniel Andrei Iordan, Mădălina-Gabriela Coman, Oana-Diana Hrisca-Eva, Alexandru Stavrică-George, Alina-Claudia Gherghin, Ilie Onu

**Affiliations:** 1 Department of Individual Sports and Kinetotherapy, Faculty of Physical Education and Sport, “Dunarea de Jos” University of Galati, 800008 Galati, Romania; daniel.iordan@ugal.ro (D.A.I.); madalina.postelnicu@ugal.ro (M.-G.C.); 2 Center of Physical Therapy and Rehabilitation, “Dunărea de Jos” University of Galati, 800008 Galati, Romania; ilie.onu@umfiasi.ro; 3 Department of Biomedical Sciences, Faculty of Medical Bioengineering, University of Medicine and Pharmacy “Grigore T. Popa”, 700454 Iasi, Romania; 4 School of Biomedical Sciences, “Dunarea de Jos” University of Galati, 800008 Galati, Romania; 5 Doctoral School, “Carol Davila” University of General Medicine and Pharmacy, 050474 Bucharest, Romania; alina-claudia.mortoiu-vasinca@drd.umfcd.ro; 6 Emergency Clinical Hospital, 050098 Bucharest, Romania

**Keywords:** knee osteoarthritis, hyaluronic acid, physiotherapy, synovial fluid, rheology, viscosupplementation, rehabilitation

## Abstract

**Background:** Knee osteoarthritis (KOA) is a degenerative joint disorder marked by cartilage degradation, synovial inflammation, and altered synovial fluid (SF) rheology, resulting in pain and impaired joint function. Intra-articular hyaluronic acid (IA-HA) injections aim to restore SF viscoelasticity and improve lubrication; however, their efficacy may be potentiated when combined with physiotherapy (PT). This monocentric observational study evaluated whether the addition of a multimodal PT program to IA-HA therapy enhances SF rheologic properties compared to IA-HA alone. **Methods:** A total of 52 patients (aged 47–61) with radiographically confirmed moderate KOA (Kellgren–Lawrence grade 2) were enrolled. Patients were assigned to a pilot group (PG; *n* = 37) receiving IA-HA (Kombihylan^®^, 3 MDa) combined with a multimodal PT protocol, or a control group (CG; n = 15) receiving IA-HA alone. The PT program included ten sessions of transcutaneous electrical nerve stimulation, low-level laser therapy, therapeutic ultrasound, progressive exercise, and cryotherapy. SF samples were collected immediately after the first injection and again at six weeks, then analyzed rheologically using the Kinexus Pro+ rheometer. Viscosity parameters were assessed via steady and oscillatory shear tests. **Results:** At baseline, both groups demonstrated comparable SF viscosity profiles. After six weeks, the PG exhibited significantly higher shear viscosity values across all measured percentiles and reduced variability in rheological parameters, suggesting a more stable intra-articular milieu. Rheometric analysis indicated enhanced SF viscoelasticity, potentially mediated by reduced inflammation and stimulation of endogenous HA synthesis. In contrast, the CG showed inconsistent viscosity changes, reflecting variable responses to IA-HA monotherapy. **Conclusions:** Combining IA-HA with multimodal PT significantly improves SF rheological properties in moderate KOA patients compared to IA-HA alone. These findings support the role of mechanical stimulation in enhancing joint lubrication and homeostasis, offering a more consistent and effective approach to viscosupplementation.

## 1. Introduction

Knee osteoarthritis (KOA) is a disease that affects the entire knee joint, including cartilage, bone, synovium, ligaments, meniscus, and muscles. While once considered non-inflammatory, it is now recognized that low-grade inflammation plays a key role in the pathogenesis of KOA, potentially contributing to disease progression [1].

Symptomatic KOA affects about 10% of men and 13% of women aged 60 years or older. The number of people with symptomatic KOA is expected to rise due to population aging and the obesity epidemic. KOA has a multifactorial etiology, resulting from the interaction of systemic and local factors. Age, female sex, obesity, knee injury, repetitive joint use, bone density, muscle weakness, and joint laxity contribute to its development. These factors particularly affect weight-bearing joints. Modifying risk factors can help lower the likelihood of KOA and its progression. Advances in imaging, biomarkers, and symptom measurement are improving understanding of KOA mechanisms. This knowledge may lead to innovative pharmacologic and non-pharmacologic therapies [2].

Standard treatments include non-steroidal anti-inflammatory drugs (NSAIDs), analgesics, and slow-acting agents with chondroprotective properties, such as glucosamine, chondroitin, and intra-articular (IA) hyaluronic acid (HA) [3].

For the non-surgical treatment of KOA, patient education, lifestyle changes, and pharmacological therapy are typically the first approach medical doctors prescribe, often involving acetaminophen, topical or oral NSAIDs, or intra-articular corticosteroids. Rehabilitation usually follows when oral medications fail to control acute symptoms or when secondary pain syndromes emerge due to chronic knee dysfunction [4].

Recent guidelines for managing KOA (Table 1) highlight the application of IA-HA, physical exercise, TENS (transcutaneous electrical nerve stimulation), and laser therapy, each receiving varying degrees of endorsement. The American Academy of Orthopaedic Surgeons (AAOS) recognizes IA-HA as a moderate intervention, while the American College of Rheumatology (ACR) offers conditional support. Physical exercise, encompassing supervised activity and balance training, is strongly supported in all guidelines for its efficacy in managing symptoms and enhancing function. In contrast, TENS is weakly recommended by the AAOS, strongly contraindicated by the ACR, and included in therapeutic modalities by the American Physical Therapy Association (APTA). Laser therapy is weakly supported by the AAOS and included in APTA’s therapeutic modalities but lacks specific endorsement from the ACR [5,6,7,8].

HA is a glycosaminoglycan found in the extracellular matrix, crucial for joint lubrication and cushioning. In KOA, HA levels decline, contributing to joint pain, stiffness, and reduced mobility. Intra-articular (IA-HA) injections aim to restore the viscoelastic properties of SF, improving joint function and relieving pain. Higher molecular weight (MW) formulations tend to be more effective [9,10]. HA also has anti-inflammatory effects, reducing levels of prostaglandin E2 (PGE2), NF-κB, and matrix-degrading enzymes [11,12,13]. Rheologically, viscosupplementation enhances SF viscosity and elasticity, improving lubrication and shock absorption. Its shear-thinning nature allows for optimal joint protection at rest and smoother movement during activity. High MW and cross-linked HA formulations provide longer-lasting effects by resisting degradation in the joint.

The rheology of SF is crucial for joint function, and its properties are influenced by the presence of HA and other proteins. In KOA, the viscoelasticity of SF decreases, but intra-articular injection of HA can restore these properties, reducing pro-inflammatory cytokines and promoting tissue repair, as observed through increased collagen and fibroblast activity.

This study is a monocentric, observational study designed to evaluate the effectiveness of combining IA-HA with a multimodal physiotherapy (PT) protocol in maintaining or improving the rheological properties of SF in patients with moderate KOA.

We hypothesized that the combined treatment (IA-HA + PT) would result in significantly better rheological outcomes compared to IA-HA alone. This hypothesis is based on the assumption that PT enhances joint mobility, synovial circulation, and tissue metabolism, while IA-HA contributes to biochemical improvement in joint lubrication and shock absorption. Together, these interventions may offer a synergistic effect on SF properties.

To provide a standardized assessment of the evidence supporting the treatment plan, we have classified the levels of evidence according to the Oxford Centre for Evidence-based Medicine (OCEBM) criteria. This approach ensures clarity and facilitates comparison with other clinical guidelines

## 2. Materials and Methods

### 2.1. Materials

The viscoelastic biopolymer used in this study, Kombihylan^®^, was purchased from Rompharm^®^ (Otopeni, Romania) and administered with approval no. 11306/2020. Kombihylan is a biological matrix with a molecular weight of 3 MDa, formulated as a viscoelastic solution containing uncrosslinked hyaluronic acid (HA) obtained through bacterial fermentation of a *Streptococcus* strain. The formulation also includes chondroitin sodium sulfate (30 mg/mL), N-acetyl D-glucosamine (30 mg/mL), sodium salts (NaCl—4.3 mg/mL; NaH_2_PO_4_·2H_2_O—0.52 mg/mL; Na_2_HPO_4_·12H_2_O—5.04 mg/mL), and NaOH/H_3_PO_4_ for pH adjustment to 7.4.

A monocentric observational study was conducted between January 2020 and July 2021 in the orthopedics and physiotherapy departments of the Micromedica Clinic in Piatra Neamț. The study was approved by the Institutional Review Board (Ethics Committee) of the Micromedica Medical Clinic (approval no. 32, dated 28 January 2020) and was carried out according to the ethical principles outlined in the Declaration of Helsinki. All patients provided written informed consent before participation. An orthopedic specialist administered the product intra-articularly under sterile conditions to patients diagnosed with moderate KOA.

### 2.2. Participants

The study included 52 patients, aged between 47 and 61, divided into two groups: a pilot group (PG) with 37 patients and a control group (CG) with 15 patients. The PG received IA-HA injections combined with a multi-modal PT program, which included 10 sessions of transcutaneous electrical nerve stimulation (TENS), low-level laser therapy (LLLT), ultrasound (US), physical exercise (PE), and cryotherapy. The CG only received IA-HA injections. All patients were diagnosed with moderate KOA, classified as Kellgren–Lawrence grade 2, and had similar baseline pain severity and disability levels. Synovial fluid probes were taken immediately after viscosupplementation and again after six weeks to evaluate the rheological properties.

The total cohort had an average age of 55.9 years, with the PG averaging 55.8 years and the CG averaging 56.1 years. There were 26 females (13.5%) in the total cohort, 16 females (5.9%) in the PG, and 10 females (1.6%) in the CG. The total cohort had a mean BMI of 30.0, with the PG having a lower average BMI of 29.0 and the CG having a higher BMI of 32.3.

Regarding symptomatic knees, 35.5% of the total cohort had right knee symptoms, 44.2% had left knee symptoms, and 19.2% had symptoms in both knees. In the pilot group, right knee symptoms were present in 45.9%, left knee symptoms in 35.1%, and both knees in 18.9%. The control group showed a higher prevalence of left knee symptoms (73.3%), with only 13.3% having right knee symptoms and 20% having symptoms in both knees.

Although initial reporting suggested that both groups had similar baseline pain severity and disability levels, consistent quantitative data for these variables were not systematically recorded and are therefore not included in the final analysis. This limitation is acknowledged and discussed in the relevant section below.

Eligible patients were those diagnosed with symptomatic moderate KOA without signs of local inflammation and prior infiltrations of other viscoelastic substances or glucocorticoids in the past 12 months. Only patients with one symptomatic knee were included.

Exclusion criteria included known allergy or hypersensitivity to sodium hyaluronate or any Kombihylan^®^ ingredients, local inflammation or hydarthrosis, infections or skin conditions at the injection site, inflammatory rheumatic or systemic diseases, or known systemic bleeding disorders.

### 2.3. Procedure Steps

The study was structured in three main phases:

Phase 1 (Baseline and Intervention Initiation): Initial clinical assessment, inclusion/exclusion screening, baseline synovial fluid aspiration, and IA-HA injection (Kombihylan^®^) for both groups.

Phase 2 (Intervention Period): Seventy-two hours after the IA-HA injection, the PG initiated a multimodal PT program consisting of 10 treatment sessions administered over two consecutive weeks (five sessions per week, Monday to Friday). Each session included TENS, LLLT, ultrasound, progressive exercise, and cryotherapy, performed in the same visit, with a total duration of approximately 80–90 min. After completing this two-week intervention, no further treatments were administered until the 6-week follow-up SF aspiration, allowing us to assess the persistence of the combined therapy effects.

Phase 3 (Follow-up and Reassessment): Six weeks post-injection, SF was re-aspirated and subjected to rheological analysis.

Procedural steps (PS) were followed to perform the experiments:

PS1—Under a sterile environment, an aspiration from the osteoarthritic knee was performed (suprapatellar region, with a needle and syringe to depressurize the joint capsule). Around 2–3 mL of SF was aspirated from the knee joint to reduce post-procedural swelling, preventing the increase in intra-articular pressure.

PS2—Intra-articular infiltration of viscoelastic product Kombihylan^®^ (3 mL) was performed in the suprapatellar region using the same needle;

PS3—After the needle was removed, the patient was asked to walk for 5–10 min to “homogenize” the viscoelastic product;

PS4—An initial SF sample (less than 1 mL) was collected approximately 10 min after the viscoelastic supplementation, using a new sterile needle and syringe. This short interval allowed the injected product to be evenly distributed within the joint cavity, ensuring that the sample reflected both endogenous SF and the supplemented HA.

PS5—After 72 h, a group of 37 patients started the program of PT for 10 consecutive days (2 weeks);

PS6—6 weeks later, SF was aspirated from the knee and evaluated rheologically.

### 2.4. Physiotherapy Treatment

Multimodal PT included the following procedures:

PT1—conventional TENS for 30–40 min, two crossed channels, 100 Hz, 100 µs, symmetrical rectangular biphasic pulses (PHYSIOMED ELEKTROMEDIZIN AG, Schnaittach, Germany);

PT2—LLLT, infrared 904 nm GaAlAs probe, 3 kHz frequency, 108 mW, with a 5 Joules/point, 8 points, and a maximum of 40 Joules/application (Pagani Roland LASER IR27, Fimad Elettromedicali, Catanzaro, Italy);

PT3—8 min of US, 0.2–0.3 W/cm^2^ at 1 MHz with a 10% duty cycle (PHYSIOMED ELEKTROMEDIZIN AG, Schnaittach, Germany);

PT4—over 40 min (per session) and consisted of moderate-intensity exercise, including the following components: a 5 min warm-up on a stationary bike; static quadriceps contractions with a 7 s hold; knee extensions over a roll, also with a 7 s hold; 50 single-leg raises; 50 step-ups; calf raises performed in three sets of 10–15 repetitions; and wall squats held for 5–10 s. Neuro-proprioceptive facilitation (PNF) techniques were incorporated using four movement patterns (MPs), each repeated 2–3 times per set: MP1 (flexion–abduction–internal rotation), MP2 (extension–adduction–external rotation), MP3 (flexion–adduction–external rotation), and MP4 (extension–abduction–internal rotation). The PNF methods applied included PNF1 (contract–relax), PNF2 (hold–relax), PNF3 (reversal of antagonists), and PNF4 (repeated stretch).

PT5—15 min Cryo-push therapy (CRYOPUSH Cold Compression Therapy System, Chengdu, China).

### 2.5. Rheologic Evaluation of Synovial Fluid

Rheometry measurements were conducted using a parallel-plate geometry with the Kinexus Pro+ rotational rheometer (Malvern Instruments Ltd., Malvern, Worcestershire, UK). All tests were performed at a controlled temperature of 37 °C (±0.1 °C) using the Peltier system on the testing plate. Data were recorded using rSpace for Kinexus Pro+ software 1.7 software (rSpace Software, NETZSCH-Gerätebau GmbH, Selb, Germany); each rheological evaluation required 0.6 mL of SF. To assess the SF’s viscosity and viscoelastic properties, both steady shear and oscillatory shear tests were performed. The rheometer determined the linear viscoelastic range through a strain test at a 10 rad/s angular frequency, with deformation amplitudes ranging from 0.01% to 100%. A 1% amplitude within the linear viscoelastic range and a frequency range of 0.01–200 rad/s were used for frequency sweep tests.

The probe temperature was maintained within ±0.1 °C using a Peltier-controlled system. Before testing, the gap between the rheometer plates was set to 0.0250 mm. Rheological characterization included both viscosity measurements and oscillatory tests. The dynamic viscosity of the SF probes was evaluated at shear rates of 2, 10, and 40 s^−1^. The oscillatory test was conducted under a constant stress of 1 Pa, with a frequency sweep ranging from 0.5 to 2.5 Hz, values corresponding to the physiological frequencies observed during normal walking and running in a healthy knee joint. The dynamic rheological properties of the SF were analyzed in terms of the storage modulus (G′, elastic component) and loss modulus (G″, viscous component).

Both the CG and PG underwent two viscosity assessments. The first test (Probe 1) was performed before treatment and measured the baseline rheological properties of the SF, including shear viscosity and related parameters. The second test (Probe 2) was conducted post-treatment, allowing for a comparative analysis of the changes in SF viscosity before and after intervention.

## 3. Results

Descriptive statistics were used to summarize the mean, standard deviation, minimum, and maximum values for shear viscosity of the SF in both groups (CG and PG). To assess normality, the Kolmogorov–Smirnov–Lilliefors test was applied. Differences between Probe 1 and Probe 2 within each group were analyzed using paired *t*-tests (for normally distributed data) or the Wilcoxon signed-rank test (for non-normally distributed data). Between-group comparisons (CG vs. PG) were made using independent *t*-tests or Mann–Whitney U tests, depending on data distribution. All rheological data were analyzed using MAPLE 2022 software and TIBCO STATISTICA v.13.

All comparisons are reported with *p*-values, Cohen’s d effect sizes, and 95% confidence intervals to provide both statistical and practical significance [14]. Effect sizes were interpreted according to the most recent guidelines as small = 0.1, medium = 0.4, and large = 0.8 [15].

The sample size was determined based on preliminary data from a pilot study assessing changes in the viscoelastic properties of SF following IA-HA injection combined with PT. Assuming a moderate effect size (Cohen’s d = 0.4) for differences in dynamic viscosity at a shear rate of 10 s^−1^ between groups, a two-tailed significance level of α = 0.05, and a desired power of 80% (β = 0.20), a minimum of 44 participants was required [15]. To account for potential dropouts or incomplete data, the final sample was increased to 52 patients, with an allocation ratio of approximately 2:1 (PG vs. CG). The calculation was performed using G*Power version 3.1.9.7.

### 3.1. Control Group Database

In the following, we will present, for comparative purposes, the main aspects measured in the patients in the CG. These patients underwent two tests at 6 weeks between them.

**Figure 1:**
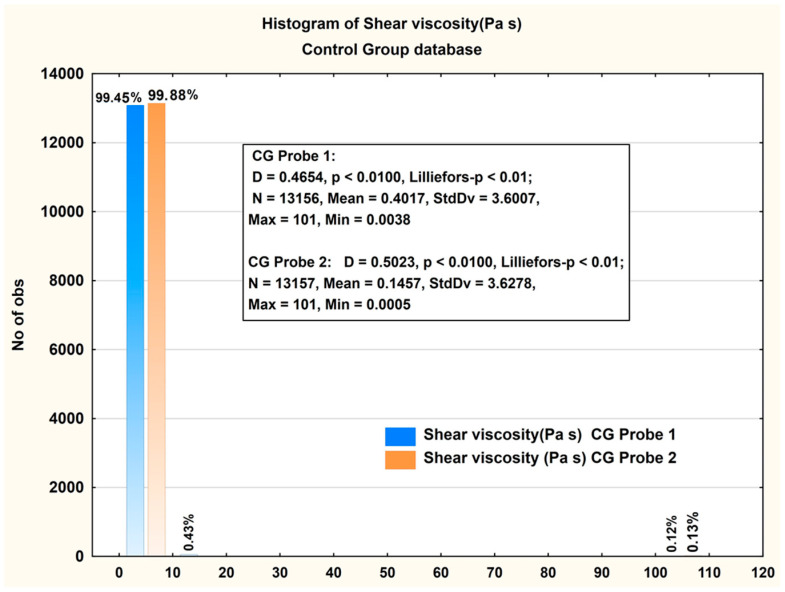
Histogram presentation of the measured viscosity in the Control Group (CG) case. Descriptive statistics of the measured viscosity are included in the figure.

Figure 1 shows histograms of the values measured for CG. The *x*-axis represents the shear viscosity values (Pa·s) measured in the CG, while the *y*-axis indicates the number of observations (data points) corresponding to each viscosity value bin. It can be seen that there is a significant difference in viscosity values between the two probes. The significance of the measured difference was verified using a *t*-test (Table 2).

**Figure 2:**
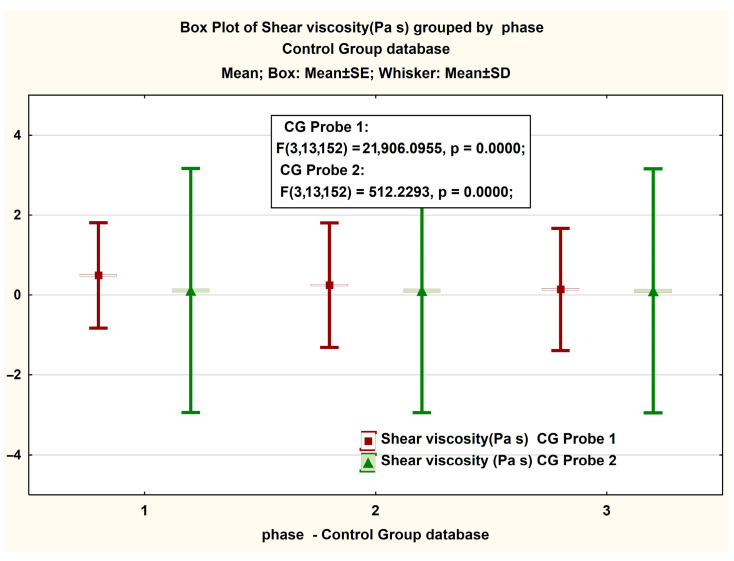
Boxplot representation of the measured viscosity in the control group (CG). The figure includes ANOVA statistical analysis results for viscosity measurements at shear rates of (1) 2 s^−1^, (2) 10 s^−1^, and (3) 40 s^−1^.

For the clarity of the presentation, we will now expose the elements measured on the CG according to the 3 phases that make up each probe for 2 s^−1^, 10 s^−1^, and 40 s^−1^(Figure 2).

In Figure 1 are presented in the form of BoxPlot graphs the measured values of the recorded viscosity for CG in both Probe 1 and Probe 2. An insignificant difference is observed between the values recorded in each phase of the test, with slightly higher values in the case of Probe 1. In Table 3, the descriptive statistical values of these measurements are presented.

In the CG, the shear viscosity of SF probes varied significantly between the two probes. Probe 1 had a higher mean viscosity (0.4017 Pa·s) compared to Probe 2 (0.1457 Pa·s). Additionally, the median for Probe 1 was 0.1628 Pa·s, while Probe 2’s median was much lower at 0.0143 Pa·s. The standard deviations for both probes were large, 3.6007 for Probe 1 and 3.6278 for Probe 2, indicating considerable variability in the viscosity measurements across probes. The 25th and 75th percentiles for Probe 1 ranged from 0.1384 Pa·s to 0.3907 Pa·s, while those for Probe 2 ranged from 0.0105 Pa·s to 0.0202 Pa·s, showing a much narrower range of viscosities for Probe 2.

These differences suggest that Probe 1 had a broader range of viscosities, potentially reflecting greater diversity in the SF characteristics among the CG probes.

**Figure 3:**
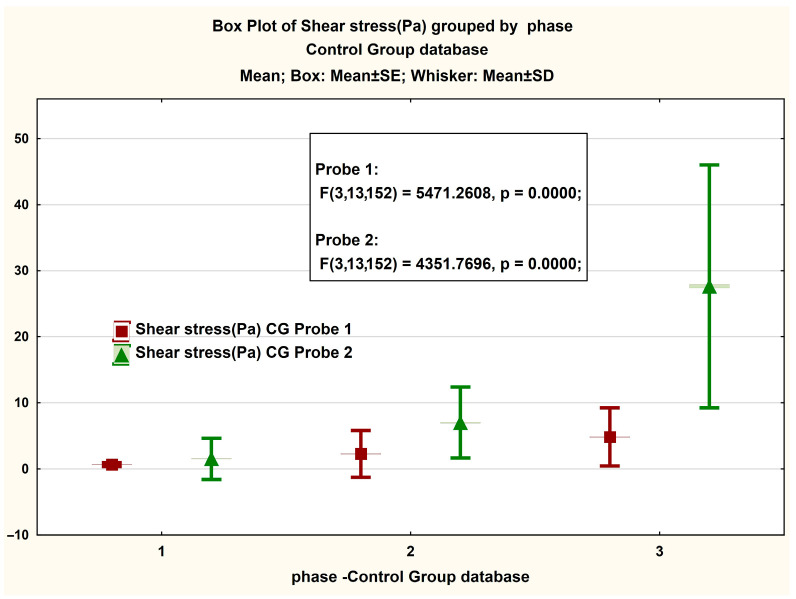
Boxplot presentation of the magnitude of the measured viscosity strength in the CG case. The ANOVA statistical analysis results for viscosity measurements at shear rates of (1) 2 s^−1^, (2) 10 s^−1^, and (3) 40 s^−1^.

**Figure 4:**
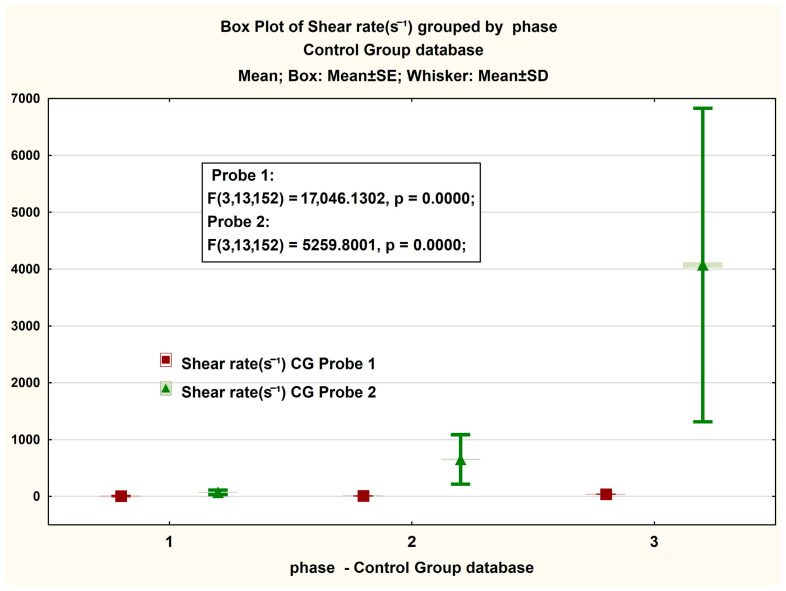
Boxplot presentation of the measured gradient—share rate in the CG case. The ANOVA statistical analysis results for viscosity measurements at shear rates of (1) 2 s^−1^, (2) 10 s^−1^, and (3) 40 s^−1^.

Figure 3 presents, in the form of BoxPlot graphs, the measured values on the magnitude of the viscosity strength recorded in both Probe 1 and Probe 2 of the GC. A non-significant difference is observed between the values recorded in phases 1 and 2 of the test, respectively, and a significant difference in the latter phase. The same interesting phenomenon is observed in the case of the box plot for the share rate (Figure 3 and Figure 4).

### 3.2. Pilot Group Database

**Figure 5:**
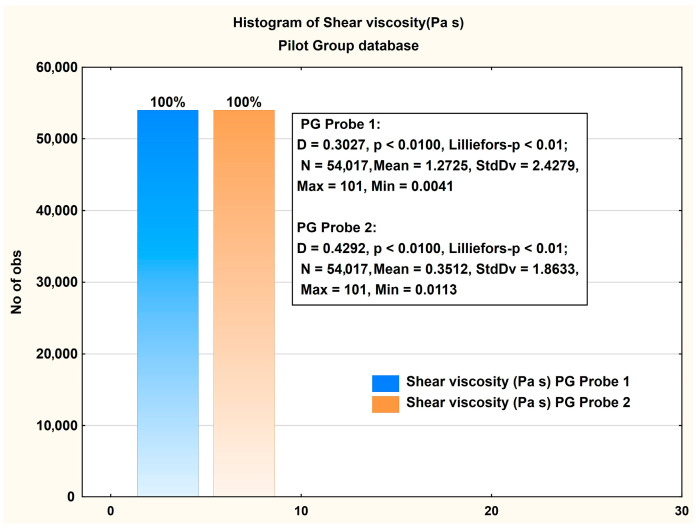
Histogram presentation of the measured viscosity for PG. Descriptive statistics of the measured viscosity are included in the figure.

In the following, we will present, for comparison, the main aspects measured on the CG. These patients were subjected to two probes with a certain time interval between them. Figure 5 shows histograms of the values measured for PG. It can be seen that there is a significant difference in the values of viscosity between the two probes.

The significance of the measured difference was verified using a *t*-test (Table 4). It is observed that the measured difference corresponds to a *p* < 0.005 level.

For the clarity of the presentation, we will now expose the elements measured on the CG according to the 3 phases that make up each probe for 2 s^−1^, 10 s^−1^, and 40 s^−1^.

**Figure 6:**
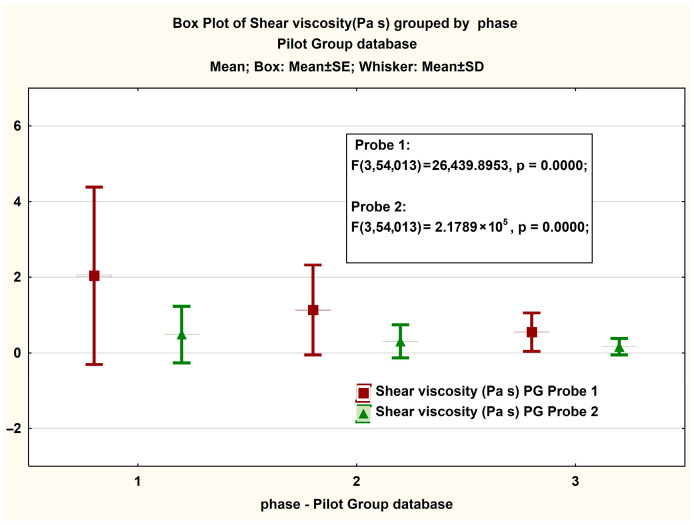
Boxplot presentation of the measured viscosity in the PG case. The ANOVA statistical analysis results for viscosity measurements at shear rates of (1) 2 s^−1^, (2) 10 s^−1^, and (3) 40 s^−1^.

In Figure 6, presented in the form of BoxPlot graphs, the measured values of the viscosity are recorded for PG in both Probe 1 and Probe 2. There is an insignificant difference between the values recorded in each phase of the test, with slightly higher values in the case of Probe 1. In Table 5, the descriptive statistical values of these measurements are presented.

In the PG, the shear viscosity of SF probes from both probes shows notable differences. Probe 1 has a significantly higher mean viscosity (1.2725 Pa·s) compared to Probe 2 (0.3512 Pa·s). The median for Probe 1 is 0.5171 Pa·s, while Probe 2’s median is much lower at 0.0894 Pa·s. The standard deviation for Probe 1 is 2.4279, which is larger than Probe 2’s 1.8633, indicating greater variability in the viscosity measurements for Probe 1. The 25th and 75th percentiles for Probe 1 range from 0.3283 Pa·s to 1.5980 Pa·s, suggesting a broader distribution of viscosity values, while those of Probe 2’s percentiles range from 0.0367 Pa·s to 0.3088 Pa·s, showing a more concentrated set of values.

Overall, Probe 1 in the PG exhibited a wider range and higher viscosity values compared to Probe 2, which shows more consistency but at a lower viscosity. This difference highlights the greater variability and higher viscosity in Probe 1 measurements within the PG.

**Figure 7:**
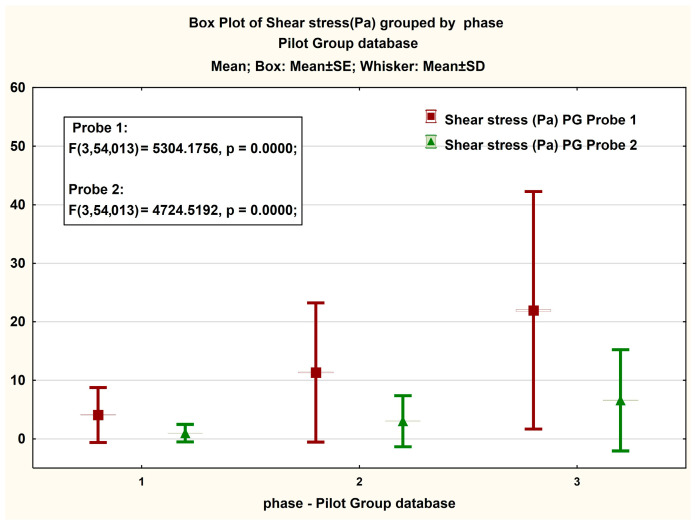
Boxplot presentation of the magnitude of the measured viscosity strength in the PG case. The ANOVA statistical analysis results for viscosity measurements at shear rates of (1) 2 s^−1^, (2) 10 s^−1^, and (3) 40 s^−1^.

**Figure 8:**
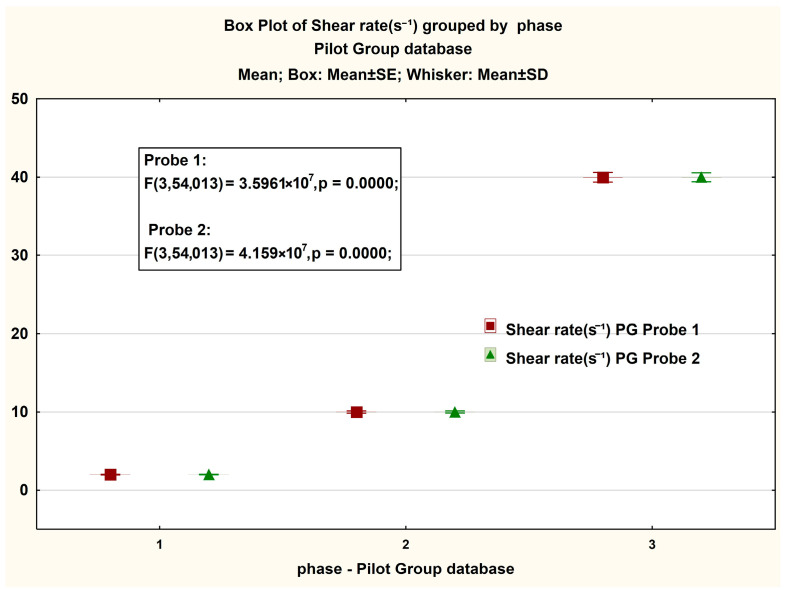
Boxplot presentation of the gradient size—share rate measured in the PG case. The ANOVA statistical analysis results for viscosity measurements at shear rates of (1) 2 s^−1^, (2) 10 s^−1^, and (3) 40 s^−1^.

In Figure 7, presented in the form of BoxPlot graphs, are the measured values on the magnitude of the viscosity strength recorded in both Probe 1 and Probe 2 for PG. We observe significant differences between the values recorded in the case of phases 2 and 3 of the test, respectively an insignificant difference in the case of phase 1. An interesting phenomenon is also observed in the case of the Box plot representation for the share rate, in which the differences between phases for each probe are significant (Figure 8).

Between-group comparisons for dynamic viscosity at a shear rate of 10 s^−1^ showed a statistically significant difference (*p* = 0.03) with a medium effect size (Cohen’s d = 0.45)**,** indicating a clinically relevant difference. In contrast, the difference in static viscosity at 1 s^−1^ was statistically non-significant (*p* = 0.12) with a small effect size (Cohen’s d = 0.15)**,** which is considered not clinically relevant and is therefore not discussed further

## 4. Discussion

This study aims to evaluate whether the combination of IA-HA and PT more effectively preserves or enhances the rheologic properties of SF in patients with moderate KOA compared to IA-HA alone. We hypothesize that integrating a multimodal PT approach with IA-HA therapy offers a superior management strategy by addressing both biochemical and mechanical factors contributing to joint degeneration.

The results support this hypothesis. Patients in the PG who received IA-HA combined with PT demonstrated significantly higher SF shear viscosity values across all measured percentiles compared to the CG, which received IA-HA alone. This suggests that the combination therapy more effectively maintains or improves SF rheology.

For Probe 1, the PG showed a notably higher mean viscosity (1.2725 Pa·s) compared to the CG (0.4017 Pa·s). Median values followed a similar trend (0.5171 Pa·s vs. 0.1628 Pa·s). Moreover, PG exhibited reduced variability (SD: 2.4279 Pa·s) versus CG (3.6007 Pa·s), indicating a more consistent response to treatment. The 25th and 75th percentiles were also higher in PG (0.3283 Pa·s and 1.598 Pa·s) compared to CG (0.1384 Pa·s and 0.3907 Pa·s), suggesting improved SF homogeneity and function.

Probe 2 yielded comparable findings. PG demonstrated higher mean (0.3512 Pa·s) and median (0.0894 Pa·s) viscosity values than CG (0.1457 Pa·s and 0.0143 Pa·s, respectively). Again, PG showed more stable outcomes, with a lower standard deviation (1.8633 Pa·s) than CG (3.6278 Pa·s). The interquartile range in PG (0.0367 Pa·s to 0.3088 Pa·s) exceeded that of CG (0.0105 Pa·s to 0.0202 Pa·s), reinforcing the observation that IA-HA combined with PT enhances SF rheologic quality.

It is important to note that the present findings are based solely on the use of high-molecular-weight HA (3 MDa). Evidence from comparative studies indicates that low-molecular-weight HA (<1 MDa) may provide symptomatic relief, but its effects are generally less consistent and shorter in duration compared to HMW-HA formulations. Meta-analyses suggest that HMW-HA is associated with greater improvements in pain and function in KOA patients, while LMW-HA demonstrates more variable outcomes [15]. Therefore, the results of this study should not be directly extrapolated to low-molecular-weight HA products [16].

An additional methodological consideration relates to the timing of the initial SF aspiration. In this study, samples were collected approximately 10 min after the intra-articular injection of HA, rather than immediately, in order to allow for homogeneous distribution of the product within the joint. The small aspirated volume (<1 mL) was intended to avoid significantly altering the intra-articular concentration of HA. Therefore, the first sample should be interpreted as representing the mixed composition of endogenous SF and exogenous HA, serving as a rheological baseline rather than a direct measure of the HA’s long-term biological effects [17].

Safali et al. [18] compared the effects of a single 60 mg IA-HA injection with three 30 mg injections administered over 12 months in patients with KOA. Both regimens improved pain and function, but the triple low-dose protocol showed significantly better results. Improvements were more notable in WOMAC, VAS, and Lequesne Index scores with the multiple-injection approach. This suggests that repeated lower-dose injections may offer enhanced therapeutic benefits compared to a single high dose. The findings support tailored IA-HA dosing strategies for more effective KOA symptom management [18].

Webner et al. (2021) [19] conducted a systematic review of 109 studies on FDA-approved IA-HA treatments for KOA. Their analysis found that Hylan G-F 20, a high MW formulation, consistently outperformed both placebo and lower MW options like Hyalgan and Supartz. Medium MW products such as Orthovisc showed mixed results, sometimes matching Hylan G-F 20 in efficacy, but not consistently. The most effective formulations identified were BioHA and Hylan G-F 20, based on improvements in VAS and WOMAC scores at 1, 3, 6, and 12 months. These findings indicate that the molecular weight of IA-HA plays a key role in treatment effectiveness, with higher MW preparations generally offering better clinical outcomes [19]

O’Neill and Stachowiak (1996), along with Oates et al. (2002), demonstrated that SF in OA exhibits rheopectic behavior, where viscosity increases over time at constant shear rates, especially at lower temperatures [20,21]. In OA, HA concentration decreases, causing SF to lose its non-Newtonian properties and become less viscous, which compromises its lubricating function [22]. Schurz and Ribitsch found that pathological SF has both lower viscosity and reduced variability compared to normal SF, correlating directly with HA depletion [23]. This decline in viscosity leads to weakened viscoelasticity and contributes to joint degeneration through a destructive feedback loop. SF, as a biological viscoelastic material, behaves differently under varying load conditions—acting viscously during walking and more elastically at higher motion frequencies like running [24,25,26,27,28].

The viscoelastic behavior of SF is described by the elastic modulus (G′) and viscous modulus (G″), where G′ reflects energy storage and G″ represents flow resistance under deformation. In healthy SF, G″ exceeds G′ at low frequencies, indicating a viscous nature, but at higher frequencies, G′ becomes dominant, showing increased elasticity [27]. In pathologic SF, particularly in KOA, G′ is significantly reduced, signaling compromised elastic function [16,28]. IA-HA helps restore SF’s viscoelasticity by increasing HA levels, reducing inflammation, and enhancing collagen and fibroblast activity. HA also supports joint lubrication through its biomechanical and gel-forming properties, maintaining the functional integrity of the extracellular matrix in articular cartilage [17,29,30].

Bhuanantanondh et al. (2010) [31] analyzed the rheological properties of OA SF using advanced rheometers and found that OA SF exhibits non-Newtonian shear thinning behavior. They also observed rheopectic behavior at 37 °C, where viscosity increased over time under constant shear. Significant intra-patient variability in SF viscosity and viscoelasticity highlighted the heterogeneity of OA-affected joints. Among the viscosupplements tested, cross-linked and higher molecular weight formulations showed superior viscosity and viscoelasticity compared to non-cross-linked or lower MW options. These findings reinforce the importance of molecular weight and cross-linking in enhancing the therapeutic performance of viscosupplements in OA treatment [31].

Anadere et al. (1979) [32] examined the viscoelastic properties of SF and HA solutions at 23 °C, finding that all exhibited non-Newtonian flow behavior influenced by HA concentration. SF with higher HA levels or from joints with degenerative diseases showed greater elasticity (G′ > G″), while SF from traumatic or rheumatic arthritis had reduced G′ and G″, especially sensitive to joint effusions. Measurements were made at 2 Hz using a Kinexus Ultra rheometer to simulate rapid joint movement [32]. Balazs et al. (2004) further showed that G′ and G″ vary with deformation frequency, with a crossover point where the fluid behavior transitions [33]. Below this point, SF acts more viscously, and above it, more elastically, indicating that HA’s mechanical response is frequency-dependent and critical for joint protection under different motion conditions [34].

Multimodal therapy in moderate KOA aims to simultaneously address the multiple mechanisms involved in pain, inflammation, and functional degradation of the knee joint. By combining procedures such as TENS, US, LLLT, PTE, and cryotherapy, a synergistic effect is aimed at improving the clinical and rheologic outcomes of SF. Previous studies have shown that these methods contribute significantly to the relief of symptoms and slow disease progression [35,36,37].

TENS is a widely used method of electrotherapy with analgesia as its main effect. TENS uses monophasic or biphasic pulsed rectangular currents that selectively activate large-diameter nonnociceptive afferents (A-β), thereby helping to reduce the sensitivity and activity of nociceptors at the segmental level. Clinical experience suggests that TENS is effective in chronic pain control and is a valuable adjunct to pharmacotherapy in acute pain management because it helps avoid overdose [37,38,39].

TENS currents reduce the levels of proinflammatory cytokines in the blood after each treatment session, thus contributing to pain control through a mechanism distinct from those previously described by decreasing the concentration of IL-6 [39]. Zhou et al. demonstrated that TENS has potential regulatory factors in increasing extracellular matrix synthesis, cell proliferation, and differentiation, and, under in vitro conditions, can be considered as a tissue engineering method to improve the cartilage repair process [40].

LASER therapy, particularly LLLT, is widely used in rehabilitation medicine due to its biostimulating, anti-inflammatory, and analgesic properties. It is characterized by monochromaticity, polarization, and coherence, allowing it to penetrate tissues effectively [41]. LLLT promotes the local production of proteolytic enzymes, histamine, serotonin, and bradykinin, and centrally increases endorphin levels, reducing pain perception. It also improves microcirculation, helps restore cellular ionic balance, and supports tissue healing [42]. Studies, such as those by Hegedus et al. (2009) [41], show that LLLT improves knee flexion and reduces pain in KOA patients for up to two months post-treatment. In one study, 27 patients received LLLT twice weekly for four weeks, showing improvements based on WOMAC and Lequesne index scores [41]. Dombrowski et al. (2018) [42] found that LLLT reduces inflammatory markers and lymphocytes while modulating nociceptor sensitivity and enhancing mitochondrial function. Furthermore, it decreases collagenase activity, stimulates collagen and myofibroblast synthesis, and aids in protecting and regenerating articular cartilage [42].

US is used in PT for the treatment of musculoskeletal conditions, with a high safety profile and beneficial therapeutic effects. They generate compressions and decompressions that improve metabolic exchange and microcirculation, reduce pain through muscle relaxation, and influence peripheral nerve conduction [43,44]. US not only relieves symptoms but can also stimulate joint cartilage repair. Recent studies show that the US promotes collagen formation and regulates inflammatory responses and has biostimulatory effects on fibroblasts. Low-intensity pulsed US, used at 0.2 W/cm^2^, altered cytokine production and influenced genes involved in tissue healing [43,44,45]. Pulsed US also favors chondrogenesis, stimulates the secretion of type II collagen and proteoglycans, and accelerates cartilage repair, having a positive effect in osteochondral disorders, including by reducing long-term degeneration [45,46].

PE is essential for preventing loss of joint function, increasing muscle strength, improving lower limb stability, and improving coordination. Their main benefits include improving physical function and quality of life. To be effective in controlling chronic inflammation, exercise should be performed for at least 150 min per week at moderate intensity or 75 min at vigorous intensity [47]. PT is essential for managing the symptoms of KOA and improving quality of life. Regular exercise helps prevent joint degradation, maintaining joint mobility and muscle tone. It helps to increase joint stability and coordination in patients with KOA [48,49]. Most clinical guidelines recommend PE as part of treatment, along with patient education and body weight management [3,4,5,6,7].

Cryotherapy is an effective, safe, affordable, and easily applied technique for managing inflammation, edema, and pain. A recent review of 58 studies (2000–2018) on its use in musculoskeletal disorders found that cryotherapy reduces pain, improves ROM, and increases patient satisfaction with minimal adverse effects [50]. It has proven effective in both surgical recovery and acute or chronic pain management. In animal models with induced KOA, cryotherapy has been shown to reduce synovial inflammation by decreasing leukocyte migration and lowering inflammatory cytokine concentrations in the knee joint. This makes cryotherapy a promising alternative to pharmacological treatments, as it can manage inflammation and improve clinical outcomes without the side effects often associated with anti-cytokine drugs [51,52].

In the current study, the viscosity of SF varied significantly between patients with KOA, highlighting the influence of factors like inflammation, joint geometry, and disease history on SF rheology. In cases treated with HA, the viscosity increased, indicating better distribution of HA in SF components, which was further stimulated by movement. PT improved the organization of proteins and polysaccharides in SF, leading to a more homogeneous distribution and slightly reduced elastic modulus [35,36,53].

Several limitations of the study need to be taken into account. The moderate probe size (52 patients) may influence the generalizability of the results, and the follow-up duration of six weeks does not allow conclusions on long-term effects. Also, the study did not include a detailed clinical assessment of symptoms such as pain and joint function, and did not have a placebo group. In addition, the use of a single formulation of HA (Kombihylan^®^) does not allow comparison with other products of different molecular weights. These limitations highlight the need for future studies with larger probe sizes, extended follow-up periods, and more detailed clinical evaluations to confirm and extend our findings.

Another limitation of this study is the absence of systematically recorded baseline values for pain severity and functional disability, despite the initial assumption of group similarity. As a result, we were unable to perform statistical comparisons for these variables or include them in outcome analyses. This may limit the interpretation of the therapeutic effect, especially in terms of symptom evolution. Additionally, the higher mean BMI observed in the CG compared to the pilot group represents a potential confounding factor (“parasite variable”) that may have influenced intra-articular biomechanics or treatment responsiveness. One limitation of our study is the sample size calculation, which was based on an assumed effect size (Cohen’s d = 0.4) derived from pilot data; this assumption may influence the precision of our power estimation and should be interpreted with caution [15].

Physical well-being represents a complex interconnection between health, nutrition and physical activity, each of these components contributing to maintaining optimal body balance and improving quality of life; however, the scientific literature includes few studies that investigate exercise addiction, body perception and the use of fitness monitoring technology in an integrated way, highlighting the need for further research in this area [54,55].

## 5. Conclusions

This study demonstrates that the combination of IA-HA injections and a multimodal PT program produces clinically relevant improvements in the rheologic properties of S in patients with moderate KOA compared to IA-HA alone. The multimodal PT approach, incorporating TENS, LLLT, US, progressive physical exercise, and cryotherapy, contributed to enhanced SF viscosity and viscoelasticity, suggesting improved joint lubrication and biomechanical function.

Patients receiving both IA-HA and PT showed greater stability in SF viscosity measurements after six weeks, whereas patients receiving IA-HA alone exhibited higher variability. These findings indicate that PT may augment the therapeutic effects of viscosupplementation by promoting synovial circulation, reducing inflammatory mediators, and potentially stimulating endogenous HA production.

The results highlight the importance of mechanical stimulation through PT in optimizing SF properties and joint function. Combining IA-HA and PT provides a more comprehensive approach to KOA management by addressing both biochemical and mechanical aspects of joint degradation. Future research should evaluate the long-term effects of this combined therapy on cartilage preservation, pain reduction, and functional mobility.

## Figures and Tables

**Table 1:** Guideline recommendations for IA-HA, physical exercise, TENS, and laser therapy in the management of knee osteoarthritis (KOA).

Intervention	AAOS	ACR	APTA
IA-HA	Moderate recommendation	Conditional support	Not specifically addressed
Physical Exercise	Strong recommendation	Strong recommendation	Strong recommendation
TENS	Weak recommendation	Strongly contraindicated	Included in therapeutic modalities
Laser Therapy	Weak recommendation	Not specifically endorsed	Included in therapeutic modalities

**Table 2:** Table with *t*-test results for Control Group (CG).

Probe 1 vs. Probe 2	Mean Probe 1	Mean Probe 2	*t*-Value	df	*p*	t Separ. Var. Est.	Std.Dev. Probe 1	Std.Dev. Probe 2
Shear rate viscosity (Pa·s)	1.272460	0.351179	69.96394	108,032	0.05	69.96394	2.427854	1.863274

**Table 3:** Descriptive statistics for the viscosity record for the Control Group.

	Shear Rate Viscosity (Pa·s)-CG Probe 1	Shear Rate Viscosity (Pa·s)-CG Probe 2
MEAN	0.401733589	0.145731335
MEDIAN	0.1628	0.0143
SD	3.60065591	3.62776811
VALID N	13,156	13,157
SUM	5285.2071	1917.38717
MIN	0.003763	0.0005183
MAX	Shear viscosity (Pa·s)	Shear viscosity (Pa·s)
25th% case	0.1384	0.01048
75th% case	0.3907	0.02024

**Table 4:** Table with *t*-test results for Pilot Group (PG).

Probe 1 vs. Probe 2	Mean Probe 1	Mean Probe 2	*t*-Value	df	*p*	t Separ. Var. Est.	Std.Dev. Probe 1	Std.Dev. Probe2
Shear rate viscosity (Pa·s)	1.272460	0.351179	69.96394	108,032	0.05	69.96394	2.427854	1.863274

**Table 5:** Descriptive statistics for the viscosity record for the Pilot Group.

	Shear Viscosity (Pa·s) PG-Probe 1	Shear Viscosity (Pa·s) PG-Probe 2
MEAN	1.27246037	0.351179198
MEDIAN	0.5171	0.08937
SD	2.42785361	1.86327381
VALID N	54,017	54,017
SUM	68,734.4917	18,969.6467
MIN	0.004103	0.01129
MAX	Shear viscosity (Pa·s)	Shear viscosity (Pa·s)
25th% case	0.3283	0.03667
75th% case	1.598	0.3088

## Data Availability

Data are contained within the main text of the article.
